# The effect of a single spinal manipulation on cardiovascular autonomic activity and the relationship to pressure pain threshold: a randomized, cross-over, sham-controlled trial

**DOI:** 10.1186/s12998-019-0293-4

**Published:** 2020-01-20

**Authors:** Mathieu Picchiottino, Margaux Honoré, Charlotte Leboeuf-Yde, Olivier Gagey, François Cottin, David M. Hallman

**Affiliations:** 1 Université Paris-Saclay CIAMS, 91405 Orsay, France; 2 CIAMS, Université d’Orléans, 45067 Orléans, France; 3 Institut Franco-européen de Chiropraxie (IFEC), Ivry-sur-Seine, Toulouse, France; 4 Institute for Regional Health Research, University of Southern Denmark, Odense, Denmark; 5 Centre for Musculoskeletal Research, Department of Occupational and Public Health Sciences, University of Gävle, Gävle, Sweden

**Keywords:** Manipulation vertébrale, high velocity low amplitude manipulation, HVLA, manipulation, système nerveux autonome, variabilité de la fréquence cardiaque, seuil de douleur à la pression, Spinal manipulation, High velocity low amplitude manipulation, HVLA, Manipulation, Autonomic nervous system, Heart rate variability, Pressure pain threshold

## Abstract

**Background:**

The autonomic nervous system interacts with the pain system. Knowledge on the effects of high velocity low amplitude spinal manipulations (SM) on autonomic activity and experimentally induced pain is limited. In particular, the effects of SM on autonomic activity and pain beyond the immediate post intervention period as well as the relationship between these two outcomes are understudied. Thus, new research is needed to provide further insight on this issue.

**Objectives:**

The aim was to assess the effect of a single SM (i.e. SM vs. sham) on cardiovascular autonomic activity. Also, we assessed the relationship between cardiovascular autonomic activity and level of pain threshold after the interventions.

**Method:**

We conducted a randomized, cross-over, sham-controlled trial on healthy first-year chiropractic students comprising two experimental sessions separated by 48 h. During each session, subjects received, in a random order, either a thoracic SM or a sham manipulation. Cardiovascular autonomic activity was assessed using heart rate and systolic blood pressure variabilities. Pain sensitivity was assessed using pressure pain threshold. Measurements were performed at baseline and repeated three times (every 12 min) during the post intervention period. Participants and outcome assessors were blinded. The effect of the SM was tested with linear mixed models. The relationship between autonomic outcomes and pressure pain threshold was tested with bivariate correlations.

**Results:**

Fifty-one participants were included, forty-one were finally analyzed. We found no statistically significant difference between SM and sham in cardiovascular autonomic activity post intervention. Similarly, we found no post-intervention relationship between cardiovascular autonomic activity and pressure pain threshold.

**Conclusion:**

Our results suggest that a single SM of the thoracic spine has no specific effect on cardiovascular autonomic activity. Also, we found no relationship between cardiovascular autonomic activity and pressure pain threshold after the SM. Further experimental research should consider the use of several markers of autonomic activity and a more comprehensive pain assessment.

**Trial registration:**

N° NCT03273868. Registered September 6, 2017.

## Résumé

**Contexte:**

Le système nerveux autonome interagit avec le système de la douleur. Les connaissances concernant les effets des manipulations vertébrales (MV) de haute vélocité et de faible amplitude sur l’activité autonome et la douleur induite expérimentalement sont limitées. En particulier, les effets des MV sur l’activité autonome et la douleur au-delà de la période située immédiatement après l’intervention sont sous-étudiés, tout comme la relation entre ces deux variables. De nouvelles études sont nécessaires pour mieux comprendre cette problématique.

**Objectifs:**

Le premier objectif était d’évaluer l’effet d’une MV (i.e. MV vs placébo) sur l’activité autonome cardiovasculaire. Le second objectif était d’évaluer la relation entre l’activité autonome cardiovasculaire et le seuil de douleur après les interventions.

**Méthode:**

Nous avons réalisé un essai randomisé, croisé, contrôlé par un placébo sur des sujets jeunes et en bonne santé (étudiants en première année de chiropraxie). L’étude comprenait deux sessions expérimentales séparées par 48 h. Les sujets recevaient durant chaque session, soit une MV thoracique, soit une manipulation placébo. L’activité autonome cardiovasculaire était évaluée en utilisant la variabilité de la fréquence cardiaque et la variabilité de la pression artérielle systolique. La sensibilité à la douleur était évaluée en utilisant le seuil de douleur à la pression. Les mesures étaient réalisées avant l’intervention et répétées trois fois après celle-ci (toutes les 12 min). Les participants et les collecteurs de données étaient en aveugle. L’effet de la MV était testé en utilisant des modèles linéaires mixtes. Nous avons testé la relation entre les variables autonomes et le seuil de douleur à la pression en utilisant des corrélations bivariées.

**Résultats:**

Cinquante-et-un sujets ont été inclus et quarante-et-un ont finalement été analysés. Nous n’avons pas trouvé de différence statistiquement significative entre la MV et le placébo en ce qui concerne l’activité autonome cardiovasculaire après l’intervention. Nous n’avons pas trouvé de relation post-intervention entre l’activité autonome cardiovasculaire et le seuil de douleur à la pression.

**Conclusion:**

Nos résultats suggèrent qu’une MV thoracique n’a pas d’effet spécifique sur l’activité autonome cardiovasculaire et qu’il n’y a pas de relation entre l’activité autonome et le seuil de douleur à la pression après la MV. On devrait considérer l’utilisation de plusieurs marqueurs de l’activité autonome ainsi qu’une évaluation plus complète de la douleur dans les recherches expérimentales futures.

## Background

Spinal manipulative techniques, i.e. mobilizations or high velocity low amplitude (HVLA) manipulations, are commonly used to treat musculoskeletal pain by chiropractors, osteopaths, and physical therapists [1]. Despite their common use and some clinical evidence supporting their efficacy [2–4], the mechanisms underlying these clinical effects are not really understood. The study of these potential mechanisms requires experimental research assessing body responses following the intervention. For instance, the effects of spinal manipulative techniques have been explored using biomechanical [5, 6] and neurophysiological outcomes, in the latter case studying e.g. neuromuscular response [7–9], pain sensitivity [10, 11], or autonomic mediated physiology [12–14].

The autonomic nervous system is a major part of the nervous system. It is divided into three parts: the parasympathetic nervous system, the sympathetic nervous system, and the enteric nervous system. Its ultimate responsibility is to ensure the maintenance of homeostasis by regulating cells, tissues, and function of organs [15]. The autonomic nervous system is controlled by supraspinal centers, such as the limbic system, hypothalamus, and some brainstem nuclei [15]. In general, autonomic activation can be assessed indirectly via some non-invasive markers of autonomic mediated physiology, such as heart rate variability (HRV) (i.e. the fluctuation in the time interval between adjacent heartbeats) [16], blood pressure variability [17], and skin conductance [18].

Evidence from experimental research suggests that mobilizations and HVLA manipulations may produce acute changes in autonomic activity. Indeed, three reviews of the literature reported that spinal mobilization may have a sympato-excitatory effect reflected by an immediate, statistically significant, increase in skin conductance compared to a sham procedure [12–14]. Evidence suggests also that spinal HVLA techniques may produce acute changes in skin sympathetic nerve activity [19, 20]. However, in a recent review, the assessment of the evidence suggested that spinal HVLA techniques, as compared to a sham, may have no acute effect on various markers of autonomic activity (e.g. cardiovascular autonomic activity) [14]. Nevertheless, in that review [14], the certainty of the evidence was considered to be very low to low. It is worth noting that a recent study [21], not included in the previous review [14], reported that a thoracic HVLA manipulation, compared to a sham, produced a statistically significant increase of the cardiac vagal activity during the immediate post intervention period. Thus, further high-quality research is needed and likely to change the conclusions of the previous review [14], at least in relation to the certainty of evidence. Also, most of the studies in this field of research reported only on short-term effects limited to the time of intervention or the immediate post intervention period [14]. Therefore, it is unknown whether changes in autonomic activation may occur after this period, and if so, the direction of these changes.

In addition to this possible autonomic effect, mobilizations and HVLA manipulations seem to have at least a short-term hypoalgesic effect, as shown by a decrease in sensitivity to experimentally induced pain (e.g. an increased pain threshold) [10, 11]. Pain and autonomic networks are closely connected and interact at the peripheral, spinal, midbrain, and cortical levels [22, 23]. For example, at the midbrain level, a complex network integrates both visceral and nociceptive inputs and initiates both autonomic and pain modulations [22, 23]. The periaqueductal gray matter, a key structure of this network, can orchestrate both short-lasting hypoalgesia associated with sympato-excitation and long-lasting hypoalgesia associated with vagal activation [24]. Therefore, based on the early studies showing hypoalgesic and sympatho-excitatory effects of spinal manipulative techniques, it has been proposed that some of these techniques might activate, at least in part, the descending pain inhibitory system projecting from the periaqueductal gray matter [25]. Although several randomized controlled trials have tested the effects of spinal manipulative techniques on both pain sensitivity and markers of autonomic activity [26–29], the relationship between these two supposed effects after this type of intervention is understudied. In fact, to our knowledge, the statistical relationship was tested only once in a study dealing with spinal mobilization [26]. This study reported a statistically significant positive correlation between manipulation-induced hypoalgesia and sympathetic excitation in a model including several pain and autonomic markers.

To summarize, there are gaps in the current knowledge regarding the effects of spinal manipulative techniques on autonomic mediated physiology and experimentally induced pain that make additional randomized controlled trials relevant. In particular, the effects beyond the immediate post intervention period as well as the relationship between these two outcomes (i.e. autonomic activity and experimentally induced pain) are largely unknown. Additionally, the certainty of evidence on the effects of HVLA spinal manipulation on autonomic activity is low [14]. Therefore, further studies on this technique, in particular, are relevant.

Finally, to provide the best quality evidence on the specific effect of the joint manipulative techniques using randomized controlled trials, the untreated control group should receive a sham intervention. This allows differentiating responses caused by the specific action of the supposed effective intervention to those attributable to context information (e.g. placebo responses) [30].

The aim was to assess, in a randomized sham-controlled trial on healthy young subjects, the specific effect of a thoracic HVLA manipulation on cardiovascular autonomic activity (i.e. heart rate and systolic blood pressure variabilities), measured repeatedly during the post intervention period. An additional aim was to assess the relationship between pressure pain threshold (PPT) and cardiovascular autonomic activity after the interventions.

Please note that another report deals with the assessment of the specific effect of the spinal manipulation on pressure pain threshold [31].

## Method

This report follows the CONSORT statement [32].

### Design and study procedure

**Fig. 1:**
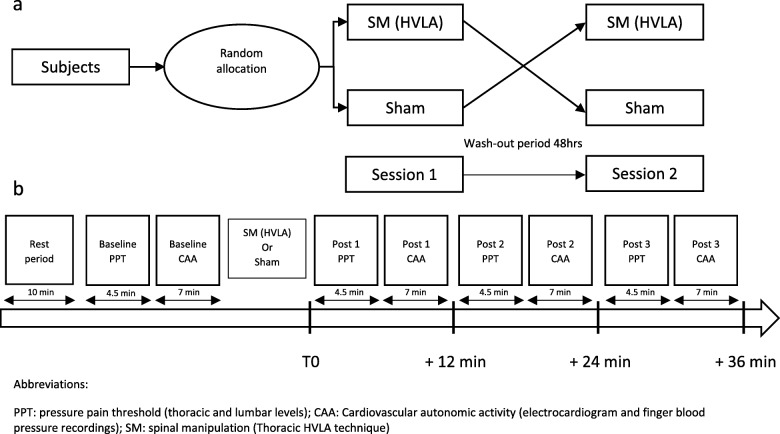
(**a**) Experimental design (**b**) Session design

We conducted a randomized, cross-over, sham-controlled trial comprising two experimental sessions separated by 48 h and scheduled at the same hour both days with each session lasting about one and a half hour. During each session, the study subject received, in a random order, either a thoracic HVLA manipulation or a sham manipulation. During each session we assessed sensitivity to experimentally induced pain (i.e. pressure pain threshold) and cardiovascular autonomic activity (i.e. HRV and systolic blood pressure variability). Measurements were performed at baseline and repeated three times (on average every 12 min) during the post intervention period. The study subjects rested for 10 min lying on their back to stabilize the cardiovascular system before baseline measurements. The experimental design is shown in Fig. 1.

### Participants

Participants were recruited among the first-year chiropractic students at the Institut Franco-Europeen de Chiropraxie, an independent chiropractic college situated in France. We chose first-year students, since they are expected to be relatively naïve to spinal manipulation and to the various types of studies dealing with this issue. Each volunteer was screened by a licensed chiropractor for eligibility criteria at the beginning of the first experimental session. Inclusion criteria were healthy volunteers, aged at least 18 years, without contra-indication to manipulative therapies. Non-inclusion criteria were pain at the time of the study, any contra-indications to spinal manipulation [33], cardiovascular or pulmonary diseases, current use of pain killers or drugs that affect autonomic physiology (e.g. beta blockers), and treatment by manipulative therapies during the previous 48 h. Other non-inclusion criteria were intake of food, caffeine, or tobacco in the hour preceding the experimentation, as well as intake of alcohol and performance of strenuous physical activity the day of the experimentation. Subjects were also asked to diminish at a maximum their use of caffeine, alcohol, tobacco and the practice of intensive physical activity during the whole trial period (i.e. from the day before the first session to the second session). After the screening process during the first session, to be included in the study, the subjects had to sign an informed consent form.

### Setting

The experiment was conducted in a laboratory room at the college from September 2017 to February 2018 and from September to October 2018. Environmental noise was kept to a minimum and the temperature was maintained at about 21 °C. The study subjects were placed on a treatment table (prone or supine position) throughout the experimental session, i.e. from the rest period to the last measurements.

### Randomization and allocation concealment

We used a drawing lot method for randomizing the order of the interventions, i.e. spinal manipulation-sham sequence or sham-spinal manipulation sequence. Allocation concealment was ensured by sealed opaque envelopes. The study subjects drew a sealed envelope from an opaque box. The sealed envelope was opened by the treating chiropractor immediately before the intervention during the first session, to ensure that ‘blinding’ to type of intervention remained unknown until this time. The study subjects were not informed that one of the interventions consisted of a sham procedure.

### Spinal manipulation and sham

The HVLA technique and the sham procedure were performed by the same licensed chiropractor during the whole trial. The study subject lay on a treatment table in a prone position for both interventions. For the *spinal HVLA technique*, the chiropractor first localized de C7 spinous process and then palpated the spinous processes up to the T5 vertebra. Then he applied a preload force with both hands placed over the transverse processes of the targeted vertebra (T5), followed by a firm thrust directed postero-anteriorly. We mainly chose this level (i.e. middle of the thoracic spine) because it is simple to perform both manipulation and a sham in this region. The *sham procedure* was applied with the subject in the same position, but the chiropractor contacted the medial border of the right scapula positioned in external rotation, applied a preload force that was followed by a thrust in the plane of the scapula-thoracic interface. This type of sham procedure has been previously used in an experimental study [34] and reported to be effective for blinding patients in a clinical trial [35]. This sham procedure did not induce spinal motion, i.e. it has a similar mechanical profile to the spinal HVLA technique but without involving spinal joints and their surrounding tissues. The chiropractor reported on a form whether audible sounds occurred or not with both the spinal HVLA and the sham techniques.

### Outcomes

#### Pressure pain threshold

To assess the effect of the thoracic HVLA technique on pain sensitivity we measured the PPT. The PPT was defined as the pressure at which the subject first indicated it became painful. This was measured in kilopascal using an Algometer type 2 (*SBMEDIC Electronics, Sweden*) with a 1 cm^2^ probe, with the study subject in the prone position. PPT was measured at two different localizations, on the paravertebral tissues (i) just right of the spinous process of the T5 vertebra, and (ii) just right to the spinous process of the L4 vertebra. An assessor, trained to assess PPT and *blinded* to the interventions, performed all measurements. The assessor increased pressure manually and perpendicularly to the skin with an application rate set at 50 kilopascal/s. The subject was instructed to press a button placed in his/her right hand to indicate when the pressure became painful (i.e. when the PPT was reached). The PPT was measured three times at each localization and at each time point (i.e. Baseline, Post 1, Post 2, Post 3). There was a 30 s rest period between each measurement. The mean of the three recordings for each time point was used in the statistical analysis, as this has been shown to be reliable in previous studies [36, 37]. Before the first session, a PPT was measured on the subject to ensure that the procedure was understood and to avoid fear or anxiety during the experimentation due to unfamiliarity with the pain stimulus (see discussion of *O’Neill* et al. [38]). The study subject could not read his/her performance level.

#### Autonomic outcome variables

##### Recording procedure

Electrocardiogram (ECG) and continuous finger blood pressure were recorded for 7 min immediately after the PPT assessment for each period of measurements (i.e. Baseline, Post 1, Post 2, Post 3). Subjects were placed in a supine position and were instructed to breathe at a pace of 0.25 Hz during the recordings, either with an auditory or visual guide, with the help of a metronome application (*Paced breathing, Trex LLC*) on a smartphone. The ECG was recorded using three electrodes connected to the PowerLab system (*ADInstruments LTD., AUS*). These three electrodes were placed on the right clavicle (earth), on the sixth left rib (positive), and on the left clavicle (negative) of each study subject. The analogous signal of the ECG was amplified with a Dual Bio Amp (*ADInstruments LTD., AUS*), connected to a PowerLab 16/35 (*ADInstruments LTD., AUS*). Noninvasive beat-to-beat blood pressure was recorded with a Finometer (*Finapres Medical Systems B.V., Netherlands*) using a finger cuff placed on the right middle finger. The Finometer was also connected to the PowerLab 16/35. The ECG and finger blood pressure signals were digitized at a sampling rate of 4000 Hz with the PowerLab device. Signals were further analyzed with LabChart on a personal computer. The assessor set up the equipment and prepared the study subjects, e.g. cleaning skin with alcohol, positioning the electrodes, the finger cuff, calibrating the Finometer. Study subjects were prevented from standing up, when they changed from the prone position (PPT assessment) to the supine position (autonomic assessment) to avoid orthostatic autonomic reflexes.

##### Data processing

A blinded assessor, who underwent a training in autonomic measures and data management, selected 5-min blocks from the 7-min recordings (i.e. ECG and blood pressure signals) for each time point (i.e. Baseline and Post 1, Post 2, Post 3) unaware of whether data pertained to spinal HVLA technique or sham (see also below). He performed (i) an automated and visual inspection of the ECG signal and (ii) a visual inspection of the blood pressure signal to detect abnormal beats, and other measurement issues (e.g. artifacts). Finally, he edited the recording using *LabChart* tools (e.g. HRV module and its beat classifier tool).

##### Heart rate variability

HRV (i.e. variability of the normal R-R intervals) was further analyzed using the HRV module in *LabChart*. This was performed in both (i) the time domain (i.e. the root mean square of the successive differences between normal heartbeats (RMSSD), and the standard deviation of the inter beat interval of normal sinus beats (SDNN)) and (ii) the low frequency (LF) and high frequency (HF) domains (i.e. LF-HRV, 0.04–0.15 Hz; HF-HRV, 0.15–0.40 Hz, LF/HF ratio) according to Task Force of the European Society of Cardiology and The American Society of Pacing and Electrophysiology [16]. Please note that the frequency analysis in the *LabChart’s HRV module* is performed with a Lomb-Scargle Periodogram, “… the Lomb method also allows for the exclusion of ectopic beats without requiring an approximated beat to be put in its place as it is perfectly capable of dealing with gaps in the data set, giving you a more accurate analysis that is less affected by ectopic or missing beats.” [39] . In short term measurements, resting SDNN is a global index of HRV and predominantly reflects vagal activity [40]. RMSSD and HF-HRV power reflect parasympathetic activity [40]. LF-HRV power may be produced by parasympathetic, sympathetic and baroreflex activities [40]. LF/HF is difficult to interpret and seems not to represent sympatho-vagal balance [40, 41], although it was included to aid comparisons with previous studies. The assessor controlled that the respiratory sinus arrythmia peak was at 0.25 Hz for each recording using the power spectrum view in *LabChart*, and if important deviations were noted, data were excluded (because this meant that the subject had not followed the paced breathing). Reliability of short term measurements of HRV at rest in healthy subjects is reported as moderate to good [42].

HRV is dependent of heart rate for both mathematical (i.e. the inverse non-linear relationships between the variability of RR intervals and heart rate) and physiological (i.e. autonomic control) reasons [43–45]. Thus, we also analyzed corrected HRV parameters as part of a sensitivity analysis (please see below). We followed the method developed by Sacha et al. [43–45], i.e. dividing the HRV parameters that have a negative relation with heart rate (e.g. LF, HF, RMSSD, SDNN) by the corresponding mean RR interval at the suitable power, to remove the mathematical bias.

##### Systolic blood pressure variability

The beat-to-beat variation in systolic blood pressure was resampled to obtain a smoother trace and to permit further spectral analysis (using fast Fourier transformation) of systolic blood pressure variability in the low frequency band (0.04–0.15 Hz) in *LabChart*. The low frequency oscillations in systolic blood pressure (LF-SBP) are proposed as a marker of the sympathetic activity to the alpha-adrenergic receptor of vasculature [17] and was used in a previous study on spinal manipulative therapies [46].

##### Other cardiovascular outcome variables

The means of heart rate, systolic blood pressure, diastolic blood pressure, and blood pressure were also calculated from each selected 5-min block.

### Blinding

#### Blinding of study subjects

The study subjects did not have access to the content of the envelope used for the randomization at any time during the whole session and were not informed of the ‘treatment’ that they would receive. At the time of the information they had been told that that the aim of the study was to assess the effect of different techniques used in manual therapies on physiological outcomes and that they would receive the same type of intervention during both sessions. Thus, we attempted to keep them naïve to the purpose of the study. Further, they were informed that the different researchers participating in this trial would not answer questions dealing with the interventions until the end of the study. They were also blinded to the recordings during the whole trial (i.e. there was no visual or auditory feedback from the algometer nor from the computer screen).

Finally, we assessed if our sham procedure had been successful to blind the study subjects. This was done using a post session questionnaire about their beliefs on the effectiveness of each intervention (HVLA manipulation and sham), to see if these were similar or if study subjects could differentiate ‘treatment’ from sham (Additional file 1). In other words, this allowed us to see if the brain-body responses to the supposed effective intervention context (e.g. placebo responses) [30] were effectively controlled by the sham procedure.

#### Blinding of the assessors during the data collection

The assessor who performed the PPT measurements left the laboratory room, when the chiropractor performed the intervention (i.e. HVLA manipulation or sham). Thus, the assessor was blinded to the intervention delivered. During experimental sessions, ECG and continuous blood pressure signals were directly recorded on a computer, and the research team had no interaction with the study subjects during these recordings.

#### Blinding of the data processing

During the treatment of the raw data, i.e. the selection of 5-min blocks of ECG and continuous blood pressure recordings and the data cleaning process (e.g. visual analysis of the data, editing of the data), the assessor was blinded to the link between the type of intervention and data. The blinding of this procedure was ensured by using transformed data file names.

Finally, the main statistical analysis (except for bivariate correlations) was also performed in a blinded manner by transforming names of the sets of data. The study groups were uncovered only at the time of data interpretation.

### Sample size

The present study assessed the effect of a thoracic HVLA manipulation on several outcomes. Therefore, it would be difficult to justify a power calculation on one particular outcome over another, as all variables had the same importance (i.e. there was no primary outcome). Instead, we determined our sample size on ‘the rule of thumb’, guided by advice of a statistician and previous literature [47]. Thus, a sample of at least 30 subjects was recommended to detect a difference between interventions, and a sample of about 50 subjects was recommended to examine a relationship with sufficient power. Therefore, our aim was to include about 50 subjects.

### Statistical analysis

SPSS Statistics for Windows, version 25 (IBM Corp., Armonk, N.Y., USA) was used for all analyses. Descriptive data are presented as frequencies for categorical variables and *mean* with *standard deviation (SD)* for continuous variables. Also, *mean (SD)* was calculated for each dependent variable for both sessions and all time points. We assessed the distribution of data with histograms and QQ plots. Dependent variables with a skewed distribution were transformed using a logarithm function (Log_10_) to achieve normality. Log transformation is usual for HRV parameters [40]. Log transformed data indicated no marked violations against normality, apart from LF-SBP.

Differences at baseline between spinal manipulation and sham were determined for each outcome variable using paired *t-tests* or Wilcoxon signed rank tests, when data were skewed. In addition, for each outcome variable, we assessed the risk of carry over effect by comparing baseline values of subjects allocated to the spinal manipulation-sham sequence to those allocated to the sham-spinal manipulation sequence using independent t-tests or Mann Whitney U tests for skewed data.

To assess the effect of the thoracic HVLA technique for each outcome variable (by comparing outcomes for the spinal manipulation and the sham) we used Generalized Linear Mixed Models. Fixed effects of the models were *Intervention* (categorical variable: spinal manipulation versus sham), *Time* (continuous variable: Baseline, Post 1, Post 2 and Post 3), and the interaction between intervention and time (*Intervention × Time*). *Time* was treated as a continuous, linear variable in all models. Quadratic effect of *Time* and its interaction with *Intervention* were added only if they improved the fit of the model (i.e. for RR intervals as outcome variable). Random intercepts were included to account for individual differences. Generalized Linear Mixed Model with a gamma distribution and a log link function was used for LF-SBP due to a skewed distribution. Within-subject correlations arising from the crossover design were taken into account in all models. Sex was found not to be a confounder and therefore excluded from the analyses. The range of age in our study subjects was too narrow to be of any importance. A statistically significant *Intervention* × *Time* interaction was interpreted as an effect of the spinal manipulation.

In a sensitivity analysis, we also analyzed corrected HRV parameters. However, results were not reported if they yielded similar conclusion as with non-corrected HRV parameters.

We visually inspected the presence of a relationship between cardiovascular autonomic outcomes and PPT (with both changes from baseline and values at each time point) using scatter plots. Also, we analyzed bivariate correlations (i.e. monotonic relationships for both changes from baseline and values at each time point) between cardiovascular autonomic outcomes and PPT. Distribution of change scores were assessed with histograms and QQ plots. We used (i) Pearson’s (parametric) or (ii) Spearman’s (non-parametric) correlation coefficient, respectively (i) if the two variables (i.e. autonomic outcomes and PPT) followed a normal distribution or (ii) if at least one of the outcome variables did not follow a normal distribution [48, 49]. Correlations were interpreted as negligible (coefficient: 0.0 to 0.3), weak (coefficient: 0.3 to 0.5), moderate (coefficient: 0.5 to 0.7), strong (coefficient: 0.7 to 0.9) or very strong (coefficient: 0.9 to 1) [48, 49].

The statistical level of significance was set at 0.05. Bonferroni corrections (dividing the alpha level by the number of tests) were applied for bivariate correlations to compensate for the risk of obtaining a significant finding by chance when performing multiple tests (i.e. Type I error).

## Results

### Participants

**Fig. 2:**
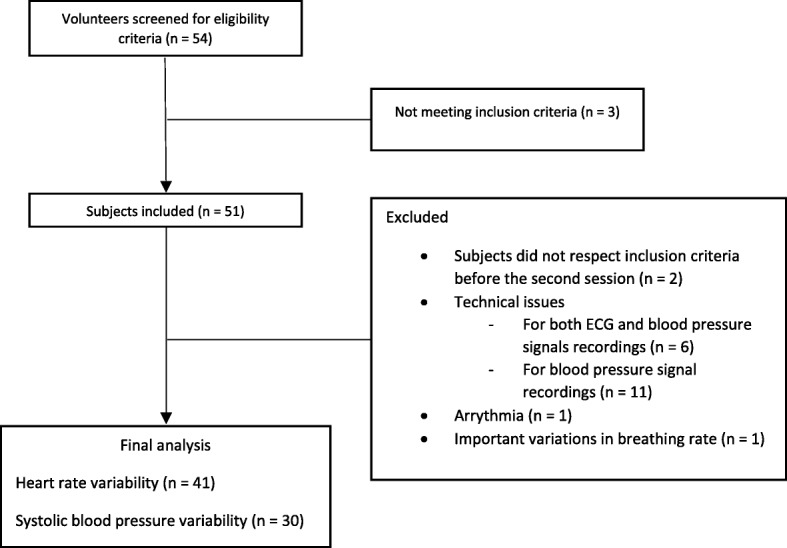
Participant flow diagram

Fifty-four volunteers were screened for eligibility criteria, 51 were included and 41 were finally analyzed. Figure 2 shows the participant flow in the study. Characteristics of the included subjects are reported in Table 1. The HVLA spinal manipulation technique produced a cracking sound coming from the spine in 90% of cases (37/41), vs. 10% (4/41) for the sham procedure. The sound produced by the sham was felt as coming from the scapula-thoracic gliding plane by the therapist.

**Table 1:** Characteristics of subjects included in the final analysis

	Number of subjects in the final analysis	Sex (Male / Female)	Age Mean (SD)	Body mass index Mean (SD)	Sequence Session 1 – Session 2 (Number of subjects)
HRV	41	19/22	19.9 (3.5)	21.6 (2.9)	SM – Sham (19)
					Sham – SM (22)
Blood pressure	30	18/12	20.2 (3.9)	21.8 (2.6)	SM – Sham (14)
					Sham – SM (16)

*Abbreviations:*

*HRV* heart rate variability; *SM* spinal manipulation (HVLA technique)

### Blinding of the sham procedure

The blinding was interpreted in two different ways.

#### First possibility

Our results suggest that the sham procedure was successful in blinding subjects in 85% of cases (Table 2. rows A, B, C, D), since subjects did not think that the sham was an ineffective procedure. Indeed, (i) 71% (Table 2. row A) of the included subjects had the same beliefs concerning the effectiveness of both interventions on the outcomes and none of them thought the sham was ineffective, (ii) 7% (Table 2. rows B, C) thought that both interventions were effective but with different levels of certainty, and (iii) 7% (Table 2. row D) thought the sham procedure was effective but did not know for the spinal manipulation. Finally, results suggest that the remaining 15% (Table 2. rows E, F, G) thought that the spinal manipulation was more effective than the sham procedure, with only 4 study subjects (Table 2. rows F, G) thinking that the sham procedure was ineffective. Among these 4 subjects, only two (Table 2. row G) thought that the spinal manipulation was effective and the sham ineffective to change the outcomes.

**Table 2:** Questionnaire about beliefs in the effectiveness of the interventions in an RCT on spinal manipulation

A	Subjects had same beliefs for SM and sham	29/41	71%
B	Subject thought that both interventions were effective and sham > SM	1/41	2%
C	Subjects thought that both interventions were effective and SM > sham	2/41	5%
D	Subjects did not know if SM was effective but thought that the sham was effective	3/41	7%
E	Subjects did not know if the sham was effective but thought that the SM was effective	2/41	5%
F	Subjects did not know if the SM was effective but thought that the sham was ineffective	2/41	5%
G	Subjects thought that SM was effective and sham was ineffective	2/41	5%

Notes

- A: 24/29 subjects thought with the same certainty that both interventions were effective and 5/29 “did not know”

- sham > SM means stronger certainty for the sham

- SM > sham means stronger certainty for the SM

*Abbreviation:*

*SM* spinal manipulation (HVLA technique)

#### Second possibility

It is also possible to consider that subjects who thought that both interventions were effective but with a stronger certainty for the spinal manipulation (Table 2. row C) were not successfully blinded. In this case the sham was probably successful in blinding subjects in 80% of cases (Table 2. rows A, B, D).

In any case, since 80% or 85% of the subjects were probably blinded and that, among them, the large majority had exactly the same beliefs regarding the effectiveness of both interventions, we can reasonably conclude that the sham procedure used in this trial was generally effective to control the brain-body responses to context information (e.g. placebo responses).

### Descriptive data

Descriptive data for each outcome variable are shown in Table 3. For both interventions (spinal manipulation and sham), mean values tended to increase over time for RRi, Log HF-HRV, Log LF-HRV, Log LF/HF, Log RMSSD, Log SDNN, and mean blood pressure, while decreasing values were observed for heart rate and HF normalized unit.

**Table 3:** Descriptive data (Mean (SD)) of all outcome variables included in an RCT on spinal manipulation for each type of intervention and at each time point

Outcomes		Spinal manipulation				Sham			
		Baseline	Post 1	Post 2	Post 3	Baseline	Post 1	Post 2	Post 3
HR (bpm)	Mean	72.0	68.1	66.9	66.7	71.6	68.4	67.0	67.4
*N* = 41	SD	11.0	9.9	9.3	9.6	9.9	9.2	9.2	9.3
RRi (ms)	Mean	856	904	921	924	857	896	917	912
*N* = 41	SD	134	136	137	140	114	115	126	128
Log HF-HRV (ms^2^)	Mean	2.971	3.096	3.205	3.194	2.981	3.086	3.107	3.141
*N* = 41	SD	0.512	0.511	0.515	0.482	0.449	0.479	0.481	0.483
HF normalized unit	Mean	66.7	65.8	62.7	63.7	67.6	65.3	63.1	61.9
*N* = 41	SD	18.0	17.0	12.7	15.5	15.2	15.3	16.1	17.6
Log LF-HRV (ms^2^)	Mean	2.610	2.739	2.940	2.898	2.603	2.758	2.831	2.876
*N* = 41	SD	0.358	0.403	0.417	0.398	0.383	0.426	0.392	0.367
Log LF/HF	Mean	− 0.361	− 0.358	− 0.265	− 0.296	− 0.378	− 0.328	− 0.276	− 0.265
*N* = 41	SD	0.398	0.389	0.259	0.319	0.341	0.338	0.342	0.375
Log RMSSD (ms)	Mean	1.647	1.733	1.787	1.781	1.651	1.717	1.740	1.754
*N* = 41	SD	0.253	0.257	0.249	0.244	0.241	0.245	0.228	0.229
Log SDNN (ms)	Mean	1.695	1.750	1.811	1.811	1.693	1.740	1.769	1.786
*N* = 41	SD	0.156	0.184	0.180	0.184	0.169	0.188	0.172	0.172
SBP (mmHg)	Mean	115.1	113.6	114.9	115.9	115.8	117.6	117.6	118.7
*N* = 30	SD	13.0	12.2	13.2	12.2	14.7	14.2	14.5	13.2
DBP (mmHg)	Mean	56.4	55.3	56.7	57.2	56.9	58.1	58.2	60.3
*N* = 30	SD	8.5	9.6	9.6	8.6	9.4	9.3	8.3	8.6
MBP (mmHg)	Mean	72.2	70.8	72.5	73.1	72.9	74.0	74.0	76.1
*N* = 30	SD	8.9	9.8	9.8	8.6	9.5	9.8	8.7	8.9
LF-SBP (mm^2^Hg)	Mean	5.907	6.954	6.858	7.420	5.637	5.782	7.233	6.949
*N* = 30	SD	4.401	5.449	4.811	5.122	3.859	4.408	5.248	5.409
Log PPT local (kPa)	Mean	2.583	2.615	2.621	2.616	2.579	2.614	2.605	2.604
*N* = 41	SD	0.161	0.170	0.166	0.169	0.166	0.151	0.167	0.178
PPT distal (kPa)	Mean	526.7	570.9	576.8	578.2	481.8	535.0	520.7	535.7
*N* = 37	SD	157.2	189.9	187.9	181.1	153.3	165.4	177.2	160.2

*Abbreviations:*

*SD* standard deviation; *HR* heart rate in beats per minute; *RRi* intervals between normal beats; *Log* logarithm with base 10; *HF* high frequency; HF normalized unit: HF/(HF + LF) x 100; *LF* low frequency; *RMSSD*: root mean square of the successive differences between normal heartbeats; *SDNN* standard deviation of the inter beat interval of normal sinus beats; *SBP* systolic blood pressure; *DBP* diastolic blood pressure; *MBP* mean blood pressure; *PPT* pressure pain threshold in kilopascal

### Baseline comparisons and carry-over effect

There were no statistically significant differences at baseline between the spinal manipulation and the sham sessions for any of the cardiovascular autonomic outcome variables. In addition, there were no statistically significant differences at each baseline (i.e. baseline spinal manipulation and baseline sham) between subjects randomized to the spinal manipulation-sham sequence and those to the sham-spinal manipulation sequence for any of the cardiovascular autonomic outcome variables except systolic blood pressure and mean blood pressure (see below). For instance, for the sham session at baseline, there was no significant difference in outcome variables between subjects who already underwent the supposed effective treatment during the first session (spinal manipulation-sham sequence) and those who started the experimentation (sham-spinal manipulation sequence). Regarding, systolic blood pressure and mean blood pressure, the difference occurred only at baseline for the spinal manipulation session, i.e. between subjects who already underwent the sham (i.e. ineffective intervention) compared to those starting the study. Thus, we can conclude that the ‘effects’ of the spinal manipulation in the first intervention period did not carry on into the next one (i.e. no carry-over effects of the spinal manipulation). The results of these different analyses are available in Additional file 2.

### Effect of spinal HVLA technique on cardiovascular autonomic activity

We found no statistically significant effect of the spinal manipulation (i.e. there were no statistically significant *Intervention × Time* interactions) for any of the cardiovascular autonomic outcomes (Table 4).

**Table 4:** Effect on cardiovascular autonomic outcomes in an RCT on spinal manipulation. Effect estimates were obtained using generalized linear mixed models

Outcomes		Intercept	Intervention (sham as reference)	Time (covariate)	Intervention × Time (sham as reference)
RRi (ms)	Estimates	856	0.2	51	4
*N* = 41	95% CI	820.7 - 892.9	− 32.3 - 32.7	37 - 65.6	− 12.9 - 21.4
	*p values*	**< 0.01**	0.989	**< 0.01**	0.626
Log HF-HRV (ms^2^)	Estimates	3.004	− 0.013	0.051	0.026
*N* = 41	95% CI	2.863 - 3.144	− 0.118 - 0.092	0.025 - 0.076	− 0.009 - 0.062
	*p values*	**< 0.01**	0.805	**< 0.01**	0.152
HF normalized unit	Estimates	67.3	− 0.8	− 1.9	0.7
*N* = 41	95% CI	63.0 - 71.8	− 5.4 - 3.8	− 3.4 - − 0.3	− 1.0 - 2.4
	*p values*	**< 0.01**	0.727	**0.018**	0.420
Log LF-HRV (ms^2^)	Estimates	2.636	− 0.001	0.089	0.018
*N* = 41	95% CI	2.518 - 2.753	− 0.095 - 0.093	0.059 - 0.117	− 0.02 - 0.056
	*p values*	**< 0.01**	0.984	**< 0.01**	0.360
Log LF/HF	Estimates	− 0.369	0.008	0.038	− 0.011
*N* = 41	95% CI	− 0.466 - − 0.271	− 0.095 - 0.111	0.006 - 0.07	− 0.048 - 0.027
	*p values*	**< 0.01**	0.877	**0.018**	0.580
Log RMSSD (ms)	Estimates	1.666	− 0.005	0.033	0.013
*N* = 41	95% CI	1.591 - 1.74	− 0.063 - 0.052	0.02 - 0.045	− 0.006 - 0.032
	*p values*	**< 0.01**	0.857	**< 0.01**	0.195
Log SDNN (ms)	Estimates	1.703	0.001	0.030	0.010
*N* = 41	95% CI	1.648 - 1.756	− 0.04 - 0.041	0.021 - 0.038	− 0.004 - 0.024
	*p values*	**< 0.01**	0.962	**< 0.01**	0.188
SBP (mmHg)	Estimates	115.6	− 0.9	1.2	− 0.7
*N* = 30	95% CI	110.2 - 120.9	− 6.4 - 4.5	0.06 - 2.3	− 2.2 - 0.8
	*p values*	**< 0.01**	0.727	**0.038**	0.389
DBP (mmHg)	Estimates	56.7	− 0.6	1.07	− 0.9
*N* = 30	95% CI	53.3 - 60.1	− 3.1 - 1.9	0.3 - 1.8	− 1.7 - 0.03
	*p values*	**< 0.01**	0.639	**< 0.01**	0.059
MBP (mmHg)	Estimates	72.7	− 0.8	1.02	− 0.75
*N* = 30	95% CI	69.2 - 76.1	− 3.7 - 2.1	0.2 - 1.7	− 1.6 - 0.1
	*p values*	**< 0.01**	0.579	**< 0.01**	0.101
LF-SBP (mm^2^Hg)	Estimates	1.557	0.003	0.050	0.050
*N* = 30	95% CI	1.252 - 1.861	− 0.272 - 0.278	− 0.02 - 0.12	− 0.048 - 0.149
	*p values*	**< 0.01**	0.981	0.161	0.313

Notes

*- Significant effects at p < 0.05 are bold faced*

*- For RRi there were a quadratic Time trend (Estimate: − 10. p < 0.01) and Intervention by quadratic Time trend (Estimate: − 0.2. p = 0.931) terms in the model*

*- For LF-SBP we used a gamma distribution with log link function*

*Abbreviations:*

*RRi* intervals between normal beats; *Log* logarithm with base 10; *HF* high frequency; HF normalized unit: HF/(HF + LF) x 100; *LF* low frequency; *RMSSD* root mean square of the successive differences between normal heartbeats; *SDNN* standard deviation of the inter beat interval of normal sinus beats; *SBP* systolic blood pressure; *DBP* diastolic blood pressure; *MBP* mean blood pressure

There were statistically significant increases in RR intervals (i.e. decrease in heart rate), log HF-HRV, log LF-HRV, log LF/HF, log RMSSD, and log SDNN over time (i.e. statistically significant effect of *Time*). Also, there were small (statistically significant) increases in systolic, diastolic and mean blood pressure over time. However, there were no statistically significant changes over time in LF-SBP. Please see Table 4 for details.

### Sensitivity analysis

A sensitivity analysis using corrected values for HF-HRV, LF-HRV, RMSSD, and SDNN for the prevailing heart rate did not change the significance of the model estimates of fixed effects (data not shown).

### Correlation between PPT and autonomic outcome variables

Visual analysis of scatter plots with PPT plotted against cardiovascular autonomic outcomes suggests neither monotonic (linear or non-linear) nor other types of relationships between the two variables.

We found mainly negligible and weak (statistically non-significant) correlations for changes from baseline to post intervention measures between cardiovascular autonomic outcomes and PPT (local and distal) after both spinal manipulation and sham interventions (Table 5). It is worth noting that there were weak and moderate (statistically significant *p* <  0.006) positive associations between changes in distal PPT and changes in both Log LF-HRV and systolic blood pressure during the sham session (Table 5).

**Table 5:** Correlation coefficients for changes from baseline between cardiovascular autonomic outcomes and PPT in an RCT on spinal manipulation

Outcomes		Log local PPT (kPa) *N* = 41			Distal PPT (kPa) *N* = 37		
		Post 1 − Baseline	Post 2 − Baseline	Post 3 − Baseline	Post 1 − Baseline	Post 2 − Baseline	Post 3 − Baseline
RRi (ms)	SM	− 0.021^†^	− 0.003	0.012	− 0.332	− 0.071	0.097
*N* = 41	Sham	− 0.237	− 0.168	− 0.098^†^	0.014	− 0.065	0.103^†^
Log HF-HRV (ms^2^)	SM	− 0.212^†^	0.073	− 0.034	− 0.067	0.007	0.070
*N* = 41	Sham	− 0.124	− 0.028^†^	− 0.184^†^	− 0.038	− 0.062^†^	0.047
HF normalized unit	SM	− 0.266^†^	− 0.049	− 0.058	− 0.037	− 0.013	0.211
*N* = 41	Sham	− 0.087	0.065	− 0.167^†^	− 0.308	− 0.026	− 0.300
Log LF-HRV (ms^2^)	SM	0.120^†^	0.126	0.077	− 0.012^†^	− 0.015	− 0.127
*N* = 41	Sham	− 0.010	− 0.205	0.134^†^	0.220	− 0.020	**0.451**
Log LF/HF	SM	0.296^†^	0.045	0.116	0.056^†^	− 0.020	− 0.200
*N* = 41	Sham	0.079	− 0.083	0.219^†^	0.262	0.036	0.376
Log RMSSD (ms)	SM	− 0.204^†^	0.161^†^	0.086^†^	− 0.102^†^	0.015^†^	0.046^†^
*N* = 41	Sham	− 0.139	− 0.006^†^	− 0.191^†^	− 0.142	− 0.041^†^	0.074
Log SDNN (ms)	SM	− 0.188^†^	0.077	− 0.081	− 0.001	0.114	− 0.024
*N* = 41	Sham	− 0.160	− 0.041	− 0.263^†^	− 0.062	− 0.145	− 0.024
SBP (mmHg)	SM	0.141^†^	0.081	0.087	0.209^†^	0.137	0.343
*N* = 30	Sham	0.165	0.247	0.336^†^	0.412	0.509	**0.581**
DBP (mmHg)	SM	− 0.010^†^	− 0.022	0.015	− 0.094	0.097	0.024
*N* = 30	Sham	0.153	0.094	0.190^†^	0.020	0.343	0.266
MBP (mmHg)	SM	0.040^†^	0.012	0.046	− 0.052	0.156	0.165
*N* = 30	Sham	0.149	0.138	0.187^†^	0.058	0.428	0.380
LF-SBP (mm^2^Hg)	SM	0.278^†^	0.142	0.164	0.062^†^	0.007	− 0.236
*N* = 30	Sham	0.181	− 0.038^†^	0.090^†^	0.379	0.043^†^	0.058

Notes

*- Statistically significant correlations at p < 0.006 (Bonferroni correction)* are bold faced

- † *Spearman’s correlation coefficients*

*- Pearson’s correlation coefficients unless contrary mention*

*- For correlations between blood pressure and distal PPT results were based (i) on 27 subjects for Post 1 - Baseline and Post 2 - Baseline and (ii) on 25 subjects for Post 3 - Baseline*

*Abbreviations:*

*SM* Spinal manipulation, *RRi* intervals between normal beats; *Log* logarithm with base 10; *HF* high frequency; HF normalized unit: HF/(HF + LF)×100; *LF* low frequency; *RMSSD* root mean square of the successive differences between normal heartbeats; *SDNN* standard deviation of the inter beat interval of normal sinus beats; *SBP* systolic blood pressure; *DBP* diastolic blood pressure; *MBP* mean blood pressure; *PPT* pressure pain threshold in kilopascal

In addition, negligible or weak (statistically non-significant) correlations were found between PPT and cardiovascular autonomic outcomes at each time point and for each type of intervention (Additional file 2).

## Discussion

### Brief summary of the findings

To the best of our knowledge, this is the first randomized sham-controlled trial assessing the effect of a spinal HVLA manipulation on both cardiovascular autonomic activity and PPT immediately and at short term (30–40 min) after the intervention, in healthy young subjects. We found no statistically significant effect of the thoracic HVLA technique on the cardiovascular autonomic activity. In other words, there was no difference on the outcomes between the thoracic HVLA technique and a valid sham procedure. In addition, we found neither monotonic (linear or non-linear) associations nor evidence of other types of relationship between cardiovascular autonomic activity and PPT after the spinal manipulation.

We noticed a decrease in heart rate over time during sessions. This was probably caused by an increase in cardiac vagal activity, as shown by the increase in log HF-HRV, log RMSSD, log SDNN. The increase in log LF-HRV over time might also indicate an increase in vagal activity. These observations may be explained by a decrease in stress after the interventions and an increase of the time spent in a recumbent position.

### Comparison with previous literature

Concerning cardiovascular autonomic control, our results are in agreement with the conclusions of a recent review of the literature on randomized sham-controlled trials, suggesting that spinal HVLA techniques may have no effect on frequency domain indices of HRV immediately after the intervention [14]. This was also the case for heart rate and blood pressure [14]. Since the certainty of evidence in this review was assessed as very low to low, it was pertinent to explore this issue again. Our findings thus strengthen this conclusion.

However, it should be acknowledged that in the current trial, the cardiovascular autonomic activity was assessed 5 min after the interventions (i.e. not during the very immediate period after the interventions), since we first measured the sensitivity to experimentally induced pain. This is different from the sham-controlled trials included in the previous review which measured HRV within the 5 min [34, 50–52] after the interventions. Our results at short term (i.e. 30–40 min) are also in accordance with another sham-controlled trial that reported no effect on the LF/HF ratio 30 min after the intervention [52].

However, a recent sham-controlled trial [21] reported a statistically significant effect of a thoracic HVLA manipulation on a time domain index of HRV (increase of RMSSD) within the 60 s following the intervention. This difference with the current study might be explained by the fact that we did not assess HRV within the minute following the intervention. It should also be noted, that their study used osteopathy students without assessing if the sham procedure was effective to blind the subjects. It was therefore uncertain whether subjects were well blinded. This might result in a performance bias and thus increase the effect size.

It is also worth noting that a recent good quality sham-controlled study testing the effect of spinal mobilization reported also no effect on HRV and PPT [29].

Further, we found no relationship between autonomic activity and pain sensitivity after the spinal HVLA technique, which is in contrast with a previous study on chronic pain patients dealing with spinal mobilization [26]. That study [26] reported a strong positive correlation between a combination of autonomic variables (skin temperature, skin blood flow, skin conductance) and a combination of pain variables (PPT, nerve tension test, pain-free grip test) using a confirmatory factor-analysis model. Therefore, the differences may be explained by the fact that we used bivariate associations between cardiovascular autonomic outcomes and PPT and that we studied healthy subjects. In addition, the joint manipulative techniques are different, the HVLA (used in our study) consists of one thrust whereas mobilization (the other study) consists of repeated oscillatory movements, which also could result in different reactions. It is also worth noting that a recent study [53] reported no relationship between an increase in sympathetic activity and symptomatic improvement after cervical mobilization in patients with cervical pain.

We found, however, a moderate (statistically significant) positive correlation between changes in systolic blood pressure and distal PPT during the sham session, which might be supported by previous literature showing an association between elevated blood pressure and a decrease in pain sensitivity [54]. However, these results should not be over interpreted, as they are found only during the sham session and on a few of the study subjects.

### Methodological consideration of the study

#### Population

As our study subjects were healthy and young, the findings might not be applicable to other populations, such as people in pain or with chronic disorders.

#### Risk of bias

We used a drawing lots method to generate the randomization as well as sealed opaque envelope for allocation concealment. Thus, the risk of *selection bias* was low. There was a roughly equal proportion of subjects allocated to the two sequences of interventions limiting the risk of period effects. The risk of carry-over effect was also low, as we used a wash-out period, and there were no results suggesting the presence of such risk.

It is difficult to blind study subjects to interventions in controlled trials dealing with spinal HVLA manipulations, since these techniques are generally well-known and easy to recognize by the general population and, in particular, by chiropractic students. Thus, including only chiropractic students might be viewed as a limitation, as they are likely to discover the true nature of both interventions (spinal manipulation and sham). Theoretically, this could increase the ‘effect’ *(performance bias*). However, we found with the post-session questionnaires that (i) the sham procedure was successful in blinding subjects and that (ii) beliefs in the effectiveness of each intervention to change the outcomes were generally similar. Hence, brain-body responses caused by the intervention context (e.g. placebo responses) [30] were probably controlled by the sham procedure. Therefore, the participation of chiropractic students did not affect the risk of performance bias, i.e. there was a low risk of *performance bias*.

During each session, physiological signals were directly recorded on a computer and further extracted and processed by a blinded assessor. Data collection for PPT was also performed by a blinded assessor. Thus, the risk of *detection bias was* low. In addition, we performed most of the statistical analysis in a blinded way (except for the correlations).

Some subjects were excluded from the final analyses because of technical issues during the experiments. In particular, blood pressure was difficult to record in these conditions (long period in a recumbent position), especially in women (e.g. loss of signal likely caused by smaller finger arteries). These exclusions reduced the statistical power, but they did not lead to any attrition bias as data from both sessions, for the remaining subjects, were analyzed. The larger number of subjects excluded for issues on blood pressure recording is briefly discussed below.

#### Technical aspects of the interventions

The study was limited to the assessment of the effect of a spinal HVLA technique applied on the middle part of the thoracic spine. Thus, the results may not be applicable to manipulation in other parts of the spine.

We used a sham procedure adopting the same physical cues as the spinal HVLA technique (i.e. preload and thrust) to improve its credibility as well as to produce similar levels of mechanical stress. This was done to control for non-specific autonomic reactions that might be caused by mechanical stress. The sham procedure was performed outside of the spinal joints complex to avoid the stimulation of the supposed ‘active ingredients’ of spinal manipulation (i.e. spinal joints and surrounding tissues). Our observations suggest, at least in part, that the sham did not produce ‘spinal’ stimulation since there was, generally, no cracking sound (and no cracking sound from the spine at all) during its execution contrary to the spinal technique.

The mechanical parameters of the interventions (e.g. preload force, peak force, and time to peak force) and thus the resulting rate of force application during the thrust might have an impact on some outcomes, as shown on the immediate neuromuscular response following HVLA manipulation [7–9]. We did not record force profiles of the interventions during the trial. Thus, we could not see if various dosages could have an impact on the outcomes. Nevertheless, the same person performed manipulation and sham to minimize variability in rate of force application between each subject.

#### Outcome variables

We assessed only cardiovascular autonomic activity, meaning that other autonomic sub-systems have not been assessed, e.g. skin sympathetic nerve activity, which was previously found to increase following mobilization with oscillatory movements as compared to a sham [12–14]. Thus, these results may not be applicable to the whole autonomic system (i.e. other autonomic sub-systems) nor to other types of manual intervention such as mobilizations. We reported several HRV parameters, as is the common use in studies dealing with this outcome variables. However, the reader should keep in mind that under these experimental conditions (i.e. short-term measurements with paced breathing) some parameters such as the HF-HRV component (marker of cardiac vagal activity) might be more reliable than others such as the LF-HRV component [42].

In addition, considering that the use of systolic blood pressure variability in this research context is still limited and that we based our conclusions on a smaller number of subjects for this particular outcome (*N* = 30), our results should be interpreted with caution and thus, replication of the results is needed.

We assessed the effect of the spinal manipulation on pain using the PPT, which explores only a limited part of the pain responsiveness [37]. Therefore, these results cannot be extrapolated to other pain aspects (e.g. affective component). The PPT assessments were performed before recording ECG and blood pressure signals, as we wanted to determine if there was an immediate hypoalgesic effect. PPT might have influenced cardiovascular autonomic activity. However, it is reasonable to think that PPT assessment had no major impact on autonomic outcomes as the pain sensation is not likely to last after the pressure stops (at least not in healthy subjects without central sensitization).

#### Relationship between cardiovascular autonomic activity and PPT

Monotonic relationships were assessed using Pearson’s or Spearman’s correlation coefficients, following previous recommendations [48, 49]. Also, we performed a visual inspection of scatter plots to ensure that there was no other types of relationship (i.e. non monotonic relationships) [49].

### Implication and perspectives

Our results do not suggest that a single spinal HVLA technique may specifically activate the descending pain inhibitory system projecting from the periaqueductal gray matter since we found (i) no effect on local and distal PPT [31] and (ii) no effect on cardiovascular autonomic outcomes. In addition, we found (iii) no relationship between PPT and autonomic responses after the HVLA technique.

Our assessment of the autonomic activation following a spinal manipulation might also allow some clinical considerations. Clinical evidence suggests that in some chronic pain condition, an increase in sympathetic activity may lead to an increase in pain [55–57] and that people with chronic pain may have an altered cardiac parasympathetic control [58, 59]. Given that spinal manipulation is often used to treat chronic pain, it seems relevant to consider if the autonomic activation following this type of intervention might be potentially harmful (i.e. increase in sympathetic activity) or beneficial (i.e. increase in cardiac parasympathetic control) for these patients. In this experimental study, we noticed no pattern of autonomic reactions after the interventions that could be considered as potentially harmful in some chronic pain conditions at short term (e.g. increase in sympathetic activity). However, this might be different in people with pain or chronic pain.

We are of the opinion that it is still reasonable to conduct experimental research on this issue because our study assessed only a limited part of the autonomic and pain systems. Further studies should then consider assessing several markers of autonomic nervous system activity (i.e. assessing various autonomic sub-systems) such as HRV and skin conductance and, very importantly, assessing several pain dimensions. Also, the potential effect of HVLA techniques applied in other parts of the spine should be considered in further studies. Sham-controlled trials should be used to control for non-specific responses and an assessment made to establish if this control procedure was effective, e.g. with the use of post-trial questionnaire. It would also be relevant to conduct such studies in a clinical context, especially on chronic pain patients who may have a disturbed cardiac autonomic (vagal) control. This would make it possible to explore if autonomic modulations after spinal manipulations are linked to health outcomes (e.g. self-reported pain) and if a course of treatments would permit to improve the cardiac autonomic (vagal) control.

## Conclusions

Our results suggest that a single HVLA manipulation of the thoracic spine has no specific effect on cardiovascular autonomic activity. Also, we found no relationship between cardiovascular autonomic activity and pressure pain threshold after the spinal manipulation. It is reasonable to conduct new experimental studies on this topic using several markers of autonomic activity with a more comprehensive pain assessment not limited to the immediate post intervention period. Even more relevant is, perhaps, to perform clinical research on people with chronic pain.

## Supplementary information

**Additional file 1.** Post session questionnaire

**Additional file 2.** Additional analyses and data

## Data Availability

The datasets used and/or analyzed during the current study are available from the corresponding author on reasonable request.

## Abbreviations

ECG: Electrocardiogram
HF: High frequency band
HRV: Heart rate variability
HVLA: High Velocity Low Amplitude
LF: Low frequency band
PPT: Pressure pain threshold
RMSSD: Root mean square of the successive differences between normal heartbeats
SDNN: Standard deviation of the inter beat interval of normal sinus beats
