# Supplementary material for: The effect of a single spinal manipulation on cardiovascular autonomic activity and the relationship to pressure pain threshold: a randomized, cross-over, sham-controlled trial
Source: Chiropr Man Therap. 2020 Jan 20;28:7. doi: 10.1186/s12998-019-0293-4 (PMC6971986; doi:10.1186/s12998-019-0293-4)
Supplement: Supplementary file 1 — Additional file 1. Post session questionnaire [file 12998_2019_293_MOESM1_ESM.pdf]

# Additional file 1

Subject ID:

Session ID:

**Post session questionnaire**

- ☐ I am definitely of the opinion that the intervention that I have received can modify the measured parameters

- ☐ I am rather of the opinion that the intervention that I have received can modify the measured parameters

- ☐ I do not know

- ☐ I am rather of the opinion that the intervention that I have received cannot modify the measured parameters

- ☐ I am definitely of the opinion that the intervention that I have received cannot modify the measured parameters

Sujet ID:

Session ID:

**Questionnaire post session**

- ☐ Je pense très fortement que l'intervention que j'ai reçue peut modifier les paramètres mesurés

- ☐ Je pense que l'intervention que j'ai reçue peut modifier les paramètres mesurés

- ☐ Je ne sais pas

- ☐ Je pense que l'intervention que j'ai reçue ne peut pas modifier les paramètres mesurés

- ☐ Je pense très fortement que l'intervention que j'ai reçue ne peut pas modifier les paramètres mesurés
