# Supplementary material for: The effect of a single spinal manipulation on cardiovascular autonomic activity and the relationship to pressure pain threshold: a randomized, cross-over, sham-controlled trial
Source: Chiropr Man Therap. 2020 Jan 20;28:7. doi: 10.1186/s12998-019-0293-4 (PMC6971986; doi:10.1186/s12998-019-0293-4)
Supplement: Supplementary file 2 — Additional file 2. Additional analyses and data [file 12998_2019_293_MOESM2_ESM.pdf]

Additional file 2

Correlation coefficients at each time point between cardiovascular autonomic outcomes and PPT in an RCT on spinal manipulation

Outcomes		Log local PPT (kPa) N = 41				Distal PPT (kPa) N = 37			
		Baseline	Post 1	Post 2	Post 3	Baseline	Post 1	Post 2	Post 3
RRi (ms)	SM	0.078	0.104	0.012	-0.077	0.194	0.088	0.065	0.059
N=41	Sham	-0.042	-0.086	-0.151	-0.057	0.088	0.064	0.009	0.042
Log HF-HRV (ms^2)	SM	-0.243	-0.202	-0.271	-0.241	0.054	-0.112	-0.031	0.032
N=41	Sham	-0.103	-0.039	-0.153	-0.124	0.076	0.223	0.078	0.073
HF nu	SM	-0.151	-0.329	-0.160	-0.177	-0.085	-0.201	-0.091	-0.049
N=41	Sham	-0.161	0.060	0.068	-0.151	-0.154	0.139	0.239	-0.108
Log LF-HRV (ms^2)	SM	-0.156	0.041	-0.269	-0.161	0.194	0.027	0.019	0.061
N=41	Sham	0.066	-0.066	-0.225	-0.003	0.257	0.162	-0.123	0.216
Log LF/HF	SM	0.172	0.307	0.105	0.164	0.088	0.170	0.084	0.025
N=41	Sham	0.210	-0.029	-0.042	0.157	0.182	-0.115	-0.227	0.119
Log RMSSD (ms)	SM	-0.174	-0.168	-0.263	-0.215	0.074	-0.093	-0.020	0.054
N=41	Sham	-0.022	-0.067	-0.177	-0.076	0.077	0.165	-0.018	0.060
Log SDNN (ms)	SM	-0.176	-0.263	-0.223	-0.207	0.101	-0.162	0.000	0.100
N=41	Sham	0.067	-0.113	-0.164	-0.050	0.151	0.128	-0.067	0.064
SBP (mmHg)	SM	0.153	0.246	0.289	0.278	-0.011	0.023	-0.042	0.200
N=30	Sham	0.144	0.012	0.090	0.154	0.014	-0.077	0.092	0.151
DBP (mmHg)	SM	-0.008	0.195	0.146	0.222	-0.135	-0.037	-0.157	-0.060
N=30	Sham	0.024	-0.126	0.051	0.101	-0.065	-0.195	-0.138	-0.083
MBP (mmHg)	SM	0.093	0.279	0.231	0.314	-0.103	-0.017	-0.115	0.039
N=30	Sham	0.152	0.006	0.162	0.203	-0.014	-0.138	-0.038	-0.007
LF-SBF (mm^2Hg)	SM	0.225†	0.164†	0.139†	0.211†	0.101†	0.252†	0.191†	0.104†
N=30	Sham	0.472†	0.398†	0.292†	0.439†	0.443†	0.292†	0.263†	0.409†

Notes:

- *Statistically significant correlations at $p < 0.006$ (Bonferroni correction)* are bold faced
- † *Spearman's correlation coefficients*
- *Pearson's correlation coefficients unless contrary mention*

Abbreviations:

SM: Spinal manipulation, RRi: intervals between normal beats; Log: logarithm with base 10; HF: high frequency; HF nu: HF/(HF + LF) ×100; LF: low frequency; RMSSD: root mean square of the successive differences between normal heartbeats; SDNN: standard deviation of the inter beat interval of normal sinus beats; SBP: systolic blood pressure; DBP: diastolic blood pressure; MBP: mean blood pressure; PPT: pressure pain threshold

Baseline comparisons and test for carry-over effect in an RCT on spinal manipulation			
	Baseline SM vs. Baseline sham	Baseline SM:<br>SM – sham sequence vs.<br>sham – SM sequence	Baseline sham:<br>SM – sham sequence vs.<br>sham – SM sequence
	Paired t test or Wilcoxon signed rank test	Independent t test or Mann-Whitney U test	Independent t test or Mann-Whitney U test
RRi (ms)	t(40) = -0.009 p = 0.992	t(39) = -1.363 p = 0.181	t(39) = -1.113 p = 0.911
N = 41		U = 166 p = 0.261	U = 207 p = 0.958
Log HF-HRV (ms^2)	t(40) = -0.195 p = 0.846	t(39) = 0.118 p = 0.906	t(39) = 0.064 p = 0.950
N = 41		U = 202 p = 0.855	U = 203 p = 0.875
HF normalized unit	t(40) = -0.390 p = 0.699	t(39) = -0.19 p = 0.985	t(39) =-0.231 p = 0.817
N = 41		U = 194 p = 0.695	U = 194 p = 0.695
Log LF-HRV (ms^2)	t(40) = 0.139 p = 0.890	t(39) = 0.210 p = 0.835	t(39) = 0.443 p = 0.660
N = 41		U = 196 p = 0.734	U = 192 p = 0.657
Log LF/HF	t(40) = 0.311 p = 0.757	t(39) = 0.037 p = 0.971	t(39) = 0.414 p = 0.681
N = 41		U = 198 p = 0.774	U = 194 p = 0.695
Log RMSSD (ms)	t(40) = -0.122 p = 0.903	t(39) = -0.334 p = 0.740	t(39) = 0.135 p = 0.893
N = 41		U = 191 p = 0.638	U = 204 p = 0.896
Log SDNN (ms)	t(40) = 0.100 p = 0.921	t(39) = 0.001 p = 0.999	t(39) = -0.480 p = 0.634
N = 41		U = 195 p = 0.714	U = 185 p = 0.530
SBP (mmHg)	t(29) = -0.275 p = 0.785	t(28) = 2.596 p = 0.015	t(39) = -0.736 p = 0.468
N = 30		U = 57 p = 0.022	U = 98 p = 0.580
DBP (mmHg)	t(29) = -0.359 p = 0.722	t(28) = 2.021 p = 0.053	t(39) = -0.540 p = 0.594
N = 30		U = 75 p = 0.131	U = 97 p = 0.552
MBP (mmHg)	t(29) = -0.477 p = 0.637	t(28) = 2.435 p = 0.022	t(39) = -0.524 p = 0.604
N = 30		U = 61 p = 0.034	U = 100 p = 0.637
LF-SBP (mm^2Hg)	Z = -0.792 p = 0.428	U = 103 p = 0.728	U = 97 p = 0.552
N = 30			

Abbreviations:

SM: spinal manipulation (HVLA technique); RRi: intervals between normal beats; Log: logarithm with base 10; HF: high frequency; HF normalized unit: HF/(HF + LF) x 100; LF: low frequency; RMSSD: root mean square of the successive differences between normal heartbeats; SDNN: standard deviation of the inter beat interval of normal sinus beats; SBP: systolic blood pressure; DBP: diastolic blood pressure; MBP: mean blood pressure;

Data for further meta-analysis

Outcomes		Baseline	Post 1	Post 2	Post 3
HR (bpm)	Mean difference	0.372	-0.262	-0.130	-0.664
N=41	SEM	1.111	0.840	0.784	0.706
RRi (ms)	Mean difference	-0.132	7.585	4.388	11.919
N=41	SEM	13.886	11.651	11.313	10.036
Log HF-HRV (ms^2)	Mean difference	-0.010	0.010	0.098	0.053
N=41	SEM	0.053	0.056	0.054	0.050
HF nu	Mean difference	-0.966	0.525	-0.425	1.801
N=41	SEM	2.479	2.756	2.487	2.666
Log LF-HRV (ms^2)	Mean difference	0.007	-0.020	0.109	0.022
N=41	SEM	0.053	0.072	0.055	0.066
Log LF/HF	Mean difference	0.018	-0.030	0.011	-0.031
N=41	SEM	0.057	0.061	0.052	0.055
Log RMSSD (ms)	Mean difference	-0.004	0.015	0.046	0.027
N=41	SEM	0.030	0.026	0.025	0.027
Log SDNN (ms)	Mean difference	0.002	0.010	0.043	0.025
N=41	SEM	0.022	0.021	0.019	0.024
LF-SBF (mm^2Hg)	Mean difference	0.270	1.172	0.008	0.599
N=30	SEM	0.667	1.103	0.751	0.793

Abbreviations:
Mean difference: Spinal manipulation – Sham; SEM: Standard error of the mean difference; HR: heart rate in beats per minute; RRi: intervals between normal beats; Log: logarithm with base 10; HF: high frequency; HF nu: HF/(HF + LF) x 100; LF: low frequency; RMSSD: root mean square of the successive differences between normal heartbeats; SDNN: standard deviation of the inter beat interval of normal sinus beats; SBP: systolic blood pressure;
